# The relationship between obesity associated weight-adjusted waist index and the prevalence of hypertension in US adults aged ≥60 years: a brief report

**DOI:** 10.3389/fpubh.2023.1210669

**Published:** 2023-10-06

**Authors:** Jiao Wang, Qing-Ye Yang, Dong-jian Chai, Yue Su, Qi-Zhi Jin, Jin-Hua Wang

**Affiliations:** Department of Cardiology, The Quzhou Affiliated Hospital of Wenzhou Medical University, Quzhou People's Hospital, QuZhou, Zhejiang Province, China

**Keywords:** hypertension, aging, obesity, WWI, NHANES, cross-sectional study

## Abstract

**Objectives:**

The main objective was to examine the relationship between weight-adjusted waist index (WWI) and the prevalence of hypertension among individuals aged ≥60 years who participated in the NHANES between 2011 and 2018 years.

**Methods:**

The data for this study were obtained from the National Health and Nutrition Examination Survey (NHANES) 2011–2018. In this population-based study, we focused on participants who were over 60 years old. Data were collected from the aforementioned survey, and the variable of interest was WWI, which was calculated as waist (cm) divided by the square root of body weight (kg). Multivariable logistic regression model was applied to calculate adjusted ORs with 95% CIs in order to explore any possible correlation between WWI and the prevalence of hypertension. Subgroup analysis were used to verify the stability of the relationship between WWI and the prevalence of hypertension. The interaction tests were also conducted in this research.

**Results:**

Results revealed that adults aged ≥60 years who were in the highest WWI quartile had significantly higher chances of developing hypertension when compared to those in the lowest quartile, after adjusting for covariates and potential confounders (*p* < 0.001).

**Conclusion:**

These findings suggest that there is a strong correlation between elevated levels of WWI and the risk of developing hypertension among older adults. As such, WWI could serve as a unique and valuable biomarker for identifying hypertension risk at an earlier stage in the older adults population.

## 1. Introduction

Hypertension, as one of the most common risk factors for cardiovascular disease, is currently defined as systolic blood pressure ≥ 130 mmHg and/or diastolic blood pressure ≥ 80 mmHg, whether for untreated patients or patients taking antihypertensive drugs (1, 2). Approximately 1 billion people worldwide suffer from the condition, making it a primary risk factor for cardiovascular and other chronic diseases (3). The prevalence of hypertension increases with age, particularly among the older adults (4). As the older adults population is more susceptible to comorbidities and the use of multiple medications that can potentially interfere with blood pressure control, hypertension-related illnesses become more burdensome with age (5). Previous studies have linked hypertension among the older adults to negative outcomes. Excess weight and abdominal obesity are important factors contributing to the development of hypertension in the older adults. Some traditional measurements such as body mass index (BMI) and waist circumference (WC) have been employed as indicators of obesity and the risk of metabolic disorders (6, 7). However, these traditional measures have limitations in a clinical setting, particularly for older individuals whose body composition and fat distribution shift due to aging.

In an effort to provide a solution to the limitations of traditional measures such as WC and BMI, a weight-adjusted waist index (WWI) has been proposed (8). Numerous studies have demonstrated the utility of WWI as a superior predictor of cardiovascular risk factors, specifically hypertension, when compared to BMI and WC (9, 10). Furthermore, WWI is more precise than either BMI or WC in predicting hypertension across various age groups, and is particularly useful in assessing risk in older adults due to its consideration of age-related loss of muscle mass (8). Several studies have shown that higher levels of WWI are associated with an increased risk of all-cause and cardiovascular mortality (10, 11), as well as cardiovascular diseases (12) such as congestive heart failure, coronary heart disease, angina/angina pectoris, heart attack, and stroke. However, so far, only one Chinese cohort study by Li et al. (13) has provided evidence of a positive association between WWI and incident hypertension. It is important to note that these associations were only observed in East Asian populations. Furthermore, this cohort study had limitations, such as a limited number of participants (650 participants) who were all aged over 60 years, and a lack of health data for individuals aged 75 years and older.

Therefore, our research is focused on investigating the potential association between WWI and the development of hypertension in the older adults population aged ≥60 years in the United States. The study utilizes data collected from the NHANES conducted between the years 2011 and 2018.

## 2. Materials and methods

### 2.1. Study populations

The data of this cross-sectional study were from the NHANES database (14). NHANES is a cross-sectional survey that employs a multistage probability sampling strategy to investigate the nutritional and health status of the noninstitutionalized civilian population of the United States (15). We focused on participants who were over 60 years old and took part in the 2011–2018 waves, comprising four consecutive survey cycles. However, we excluded individuals whose data on key variables such as blood pressure, WC, weight, and other covariates were missing. The NHANES survey protocol was approved by the NCHS Research Ethics Review Board (Protocol Number: NHANES 2011-October 26, 2017 waves: Protocol #2011-17; NHANES October 26, 2017–March 2020 Pre-Pandemic waves Protocol #2018-01.), and written informed consent was obtained from all the participants.

### 2.2. Independent variable

The independent variable under investigation for this study was WWI, which was calculated by dividing the WC in centimeters by the square root of the weight (16).

### 2.3. Outcome variables

In the Mobile Examination Center (MEC), certified examiners measured blood pressure according to ‘standardization of protocol for reducing blood pressure measurement error’ in the NHANES manuals (17). Briefly, prior to blood pressure measurement, all participants sat in a height-adjustable chair, keeping their spine straight, and sat quietly for about 5 min. Participants exposed their right arm (unless the use of the right arm was prohibited under specific conditions), placed it on the table, palms up, elbows slightly flexed. The midpoint of the right upper arm needed to be at the level of the heart. The trained examiners used a mercury sphygmomanometer to measure blood pressure three consecutive times (if the blood pressure measurement was incomplete or interrupted, the fourth blood pressure measurement could be attempted). After discarding the first blood pressure measurement record, the average value of the subsequent consecutive blood pressure measurement was the final average blood pressure record. If there was only one blood pressure reading, this blood pressure reading was regard as the final average blood pressure record. Hypertension was defined as a mean SBP ≥ 130 mmHg, or a mean DBP ≥ 80 mmHg (18). In addition, participants who meet one the following two criteria (a: self-reported history of hypertension; b: reported use of antihypertensive medications currently in the NHANES Questionnaire Data section) were also considered as hypertension (19).

### 2.4. Covariates

Based on previous research findings (20, 21), variables such as demographic characteristics, socioeconomic background, lifestyle behaviors, physical examinations, laboratory tests, and personal medical history were selected as potential confounders to assess the association between WWI and hypertension. To collect data on these variables, a standardized self-reported questionnaire and computer-assisted personal interview (CAPI) were used to examine the demographic characteristics, socioeconomic background, lifestyle behaviors, and personal medical history of the participants. Trained medical staff conducted interviews with respondents in their homes, while physical examinations and laboratory tests were carried out by trained medical personnel in mobile examination centers (MEC). For more detailed information, please refer to the following sections:

• Demographic variables
➢ Age (years, continuous variables)
➢ Gender
➢ Race
• Socioeconomic background
➢ Education level
➢ Income to poverty ratio (continuous variables)
• Lifestyle behaviors
➢ Alcohol drinking
➢ Smoking status
➢ Sedentary behavior (min/d, continuous variables)

In this study, information on sedentary behavior (SB) was obtained through self-reporting in the NHANES using the Physical Activity Questionnaire (PAD 680 item) (22). Specifically, SB was assessed with the question “How much time do you usually spend sitting on a typical day?” SB was defined as activities that do not increase energy expenditure beyond the resting level, including activities such as sitting and lying down during waking hours, working on a computer, watching TV, and engaging in other forms of screen-based entertainment (excluding time spent sleeping) (23). Participant responses were coded as the average time spent in a day over the past 30 days, with the duration of SB ranging from 0 to 1,320 min per day (24). This measurement has been widely utilized in various studies associated with NHANES (25–28).

• Physical examinations
➢ Weight (kg, continuous variables)
➢ Waist circumference (cm, continuous variables)
• Laboratory tests
➢ FBG (mg/dl, continuous variables)
➢ TG (mg/dl, continuous variables)
➢ HDL (mg/dl, continuous variables)
• Medication usage
➢ Lipid lowing drugs (yes/no)
➢ Anti diabetes medicine (yes/no)
• Personal medical history
➢ Diagnosis of diabetes (yes/no)
➢ CVD diagnosis (yes/no)
➢ Stroke diagnosis (yes/no)
➢ Arthritis diagnosis (yes/no)

Medical co-morbidities were obtained through the medical condition questionnaire section of the NHANES. The data was collected by asking participants if a medical professional had ever diagnosed them with any specific conditions. The inquiry used the following prompt, ‘Has a doctor ever indicated that you have been diagnosed with _____?’

### 2.5. Statistical analysis

Baseline characteristics of the included participants were divided into hypertension and non-hypertension groups. Continuous variables, including age, income to poverty ratio, sedentary behavior, waist circumference, TG, HDL-C, and FPG, were reported as the mean ± standard deviation (SD). Categorical variables, such as alcohol use status, smoking status, and comorbidities, were reported as numbers with percentages. Student’s t-test was used to compare the mean levels between the hypertension group and the non-hypertension group if the variable was normally distributed, otherwise, the Mann–Whitney U test was adopted. Chi-square tests were used to compare the percentages of categorical variables between different groups (29). The study perceived WWI as either a categorical variable, with cutoff values from the quartile, or a continuous variable. Then, we developed multivariable logistic regression models in order to obtain ORs with 95% CI, aiming to discover the potential correlation between WWI and the prevalence of hypertension. Three models were utilized, Model 1 with no covariates, Model 2 with age, gender, and race adjusted, and Model 3 with gender, race, age, smoking status, ratio of family income to poverty, level of education, alcohol consumption, sedentary time, physical activity, TG, HDL-C, FPG, diabetes, CVD, stroke, arthritis, blood urea nitrogen, blood creatinine lipid lowing drugs, and anti diabetes medicine adjusted. In addition, we conducted subgroup analysis stratified by income to poverty ratio, gender, level of education, race, age, alcohol drinking, sedentary behavior, diabetes diagnosis, CVD diagnosis, TG and arthritis, which were presented with Model 3. The threshold for statistical significance was set at *p* < 0.05. The statistical software programs EmpowerStats (version 5.0) and R (version 4.2.2) were utilized for all statistical analyses.

## 3. Results

Totally, 39,156 participants were enrolled, after the exclusion of individuals younger than 60 years (*N* = 13,519), missing the data on waist circumference, weight, and hypertension diagnosis (*N* = 12, 100), and missing data on covariates (level of education, income to poverty ratio, smoking status, alcohol drinking, sedentary behavior (min/d), physical activity, fasting plasma glucose [FPG] (mg/dl) and other lab measurements, diabetes diagnosis, CVD diagnosis, stroke, arthritis, lipid lowing drugs, and anti diabetes medicine; *N* = 4, 208), 9, 329 participants were included finally (Supplementary Figure S1).

### 3.1. Demographic characteristics

Supplementary Table S1 shows the demographic information pertaining to older adults participants with hypertension. It was observed that hypertensive older adults patients were predominantly female, of a more advanced age, and identified as non-Hispanic Black race. Additionally, they were more likely to have a history of smoking and drinking (*p* < 0.001) and were afflicted with a greater number of chronic conditions. Further analysis showed that individuals with hypertension experienced lower family income and educational levels. Furthermore, they had a longer period of sedentary activity, and inadequate physical activity levels.

### 3.2. Investigating the possible associations between WWI and hypertension in the older adults population

**Figure 1:**
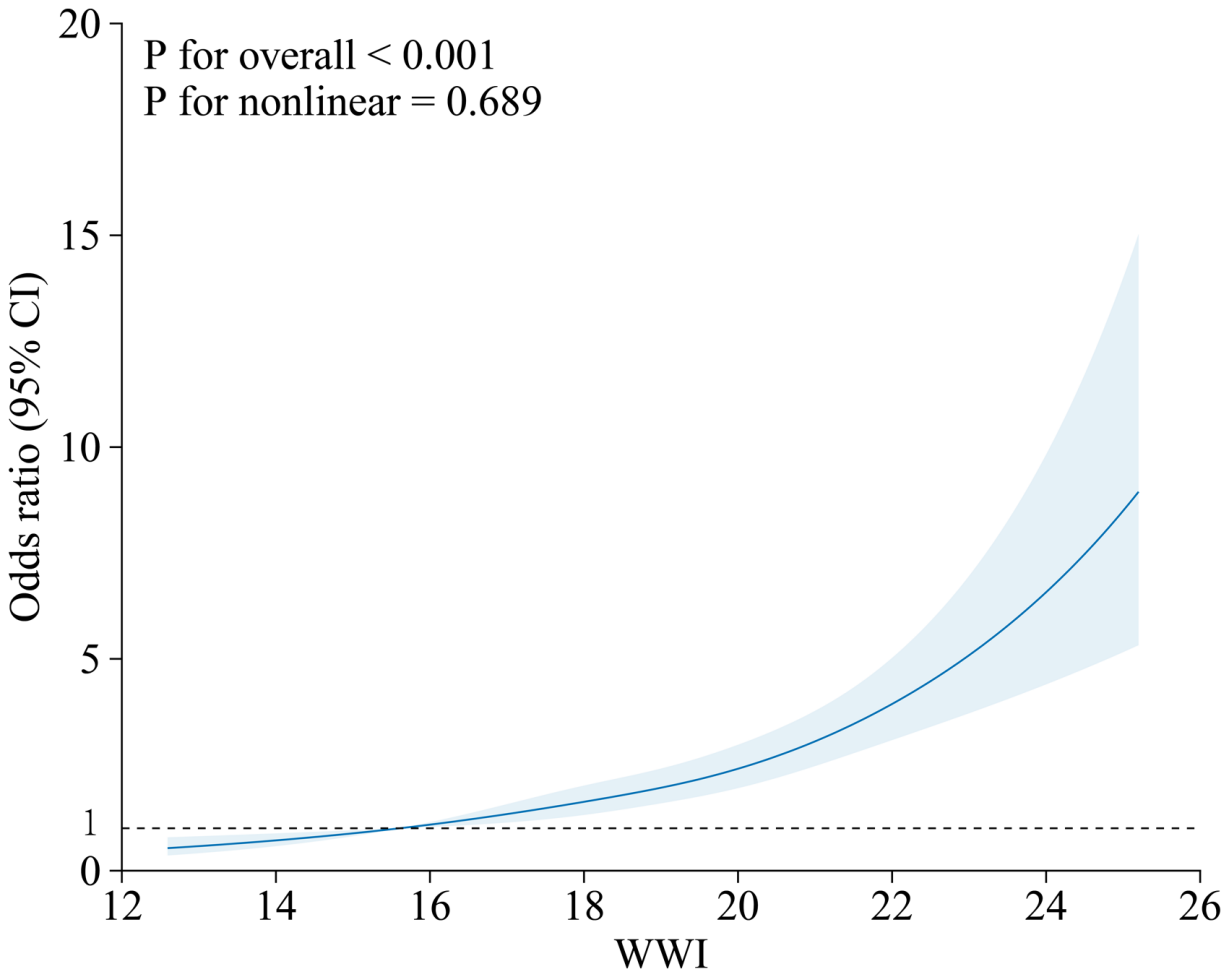
Illustrates the correlation between WWI and the prevalence of hypertension, as determined by a cubic restricted spine curve in a fully-adjusted model. The model was conducted with 4 knots at the 5th, 35th, 65th, 95th percentiles of WWI. The solid lines represent the OR that is adjusted for multiple variables including age, gender, race, education level, income-to-poverty ratio, smoking, alcohol consumption, sedentary behavior, physical activity, fasting plasma glucose, triglycerides, high-density lipoprotein cholesterol, blood urea nitrogen, blood creatinine, diabetes and cardiovascular disease diagnoses, stroke, arthritis, lipid-lowering drugs, and anti-diabetes medication. The shadow shape depict the 95% confidence interval derived from a restricted cubic spline regression. OR, odds ratio; CI, confidence interval.

It has been observed, as depicted by the data presented in Figure 1, that there exists a distinct linear correlation between WWI exposure and the prevalence of hypertension in older adults individuals. This association holds true, even after accounting for any potential elements that may have acted as confounding factors (*p* for nonlinear = 0.689 and *p* for overall < 0.001).

Table 1 displays the relationship between WWI and the prevalence of hypertension among the older adults. As a continuous variable, a higher WWI was linked with a considerable probability of hypertension according to the crude model (*p* < 0.001). The positive correlation persisted between WWI and the prevalence of hypertension in the minimally corrected model (*p* < 0.001). Moreover, the association remained statistically significant after adjusting for multiple covariates in the fully adjusted model (*p* < 0.001). In addition, patients with hypertension in the highest WWI quartile, when compared to those in the lowest quartile, had substantially higher chances of developing hypertension in all three models (Model 1: OR = 2.57, 95% CI: 2.26–2.92; Model 2: OR = 2.92, 95% CI: 2.56–3.33; Model 3: OR = 3.23, 95% CI: 1.59–6.53; all *P* for trend <0.001), pointing out the robust positive relationship between WWI and the prevalence of hypertension (Table 1). However, the statistically significant difference between quartile 2 was only detected in the crude and minimally adjusted models.

**Table 1:** Multivariable-adjusted ORs and 95% confidence intervals of WWI associated with the prevalence of hypertension.

WWI index	Model 1 OR (95% CI)	Model 2 OR (95% CI)	Model 3 OR (95% CI)
Continuous per unit increase	1.21 (1.18, 1.24)^**^	1.24 (1.22, 1.28)^**^	1.32 (1.14, 1.53)^**^
Categorical			
Quartile 1	Reference	Reference	Reference
Quartile 2	1.42 (1.24, 1.63)^**^ 1.79	1.50 (1.31, 1.72)^**^	1.59 (0.87, 2.91) 2.29
Quartile 3	(1.57, 2.04)^**^	1.89 (1.65, 2.16)^**^	(1.23, 4.26)^*^
Quartile 4	2.57 (2.26, 2.92)^**^	2.92 (2.56, 3.33)^**^	3.23 (1.59, 6.53)^**^
*p* for trend	<0.001	<0.001	<0.001

Model 1: no covariates were adjusted. Model 2: age, gender, race was adjusted. Model 3: age, gender, race, level of education, ratio of family income to poverty, level of education, smoking status, alcohol drinking, sedentary behavior, physical activity, fasting plasma glucose, triglycerides, high-density lipoprotein cholesterol, blood urea nitrogen, blood creatinine, diabetes diagnosis, CVD diagnosis, stroke, arthritis, lipid lowing drugs, and anti diabetes medicine were adjusted.

* *p* value < 0.05;

** *p* value < 0.001.

### 3.3. Subgroup analysis

**Figure 2:**
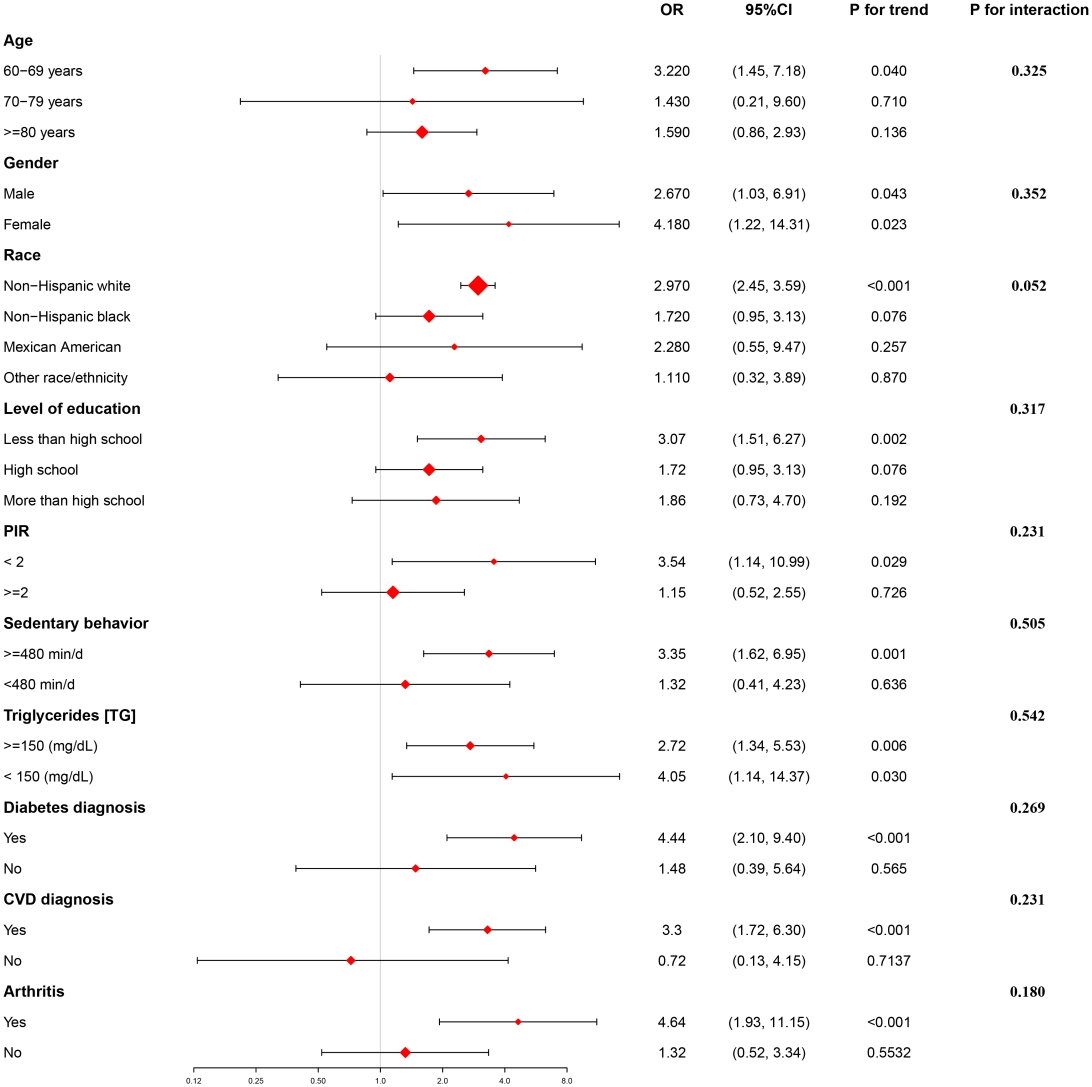
Showcases the results of a subgroup analysis that focuses on the correlation between the prevalence of hypertension in older adults individuals and WWI.

As depicted in Figure 2, we conducted a subgroup analysis. After adjusting for all confounding factors in Model 3 (refer to Table 1), stratified analysis was performed to validate the existing association between WWI and hypertension among certain participant subgroups. Notably, a stronger association was observed among those with lower than high school education, aged between 60 and 69 years, had income to poverty ratio < 2, exhibited sedentary behavior ≥480 min/d, identified as non-Hispanic white, and had underlying medical conditions such as arthritis, diabetes, and CVD. Similarly, significant associations were also observed in both males and females, and in those with TG levels above and below 150 mg/dl. Notably, there was no significant dependence on age, gender, level of education, income to poverty ratio, TG levels, sedentary behavior, diagnosis of diabetes, CVD diagnosis, and arthritis on this positive association (all *p*_interaction_ > 0.05). Overall, the analysis showed significant and robust positive associations across diverse subgroups.

## 4. Discussion

### 4.1. Summary findings

Our results showed that a high level of the WWI was associated with a higher odds of having hypertension in the older adults population. Moreover, this association remained statistically significant after adjusting for various confounding factors. Furthermore, this association was more prominent in patients aged between 60 and 69 years, those who were non-Hispanic white, had less than high school education, PIR below 2, sedentary time over 480 min/d, and were diagnosed with diabetes, CVD or arthritis.

### 4.2. Comparison with previous studies

Previously, Ostchega et al. (30) discovered that individuals classified as abdominally obese in the USA had a higher prevalence of hypertension as opposed to those who were not categorized as abdominally obese. This finding was consistent across almost all age demographics, including individuals aged 60 to 80 years old in the USA (with an odds ratio of 1.81 and a 95% confidence interval of 1.28–2.57). Crawford et al. (31) conducted a study using the GE Centricity Electronic Medical Record database, which highlighted the significant correlation between obesity and hypertension. Furthermore, the data suggested that hypertension had the highest prevalence rate (72.6%) among older patients aged over 60 with BMI of 50 or greater. The Hispanic EPESE study (32), conducted between 1993 and 1994, discovered that a BMI of ≥30 kg/m^2^ was significantly linked to measured hypertension in Mexican American older adults individuals. Our study’s results also indicated that WWI, a novel measure of central adiposity, had a positive association with the prevalence of hypertension in both crude and adjusted models. A recent study conducted in China analyzed the association between WWI and incident hypertension among a rural cohort of individuals aged 35 years or older. The results demonstrated that WWI was positively correlated with incident hypertension. However, no statistically significant correlation was found between WWI and hypertension among patients aged ≥60 years, which was inconsistent with the results of our study.

To address this issue, we compared our study to Li et al.’s study, which focused on a specific population in rural China. Unlike their study, we included a diverse sample of 6,674 older adults hypertensive patients across the United States. In contrast, Li et al.’s study only included 650 hypertensive patients aged over 60 years and did not have health data for individuals over 75 years old. The small sample size of hypertensive patients over 60 years of age in their study may have resulted in insufficient statistical power to detect a positive relationship (33). Due to ethnicities, cultural differences, diet habits, genetic variances, and lifestyle factors between Chinese and American older adults populations, we found that WWI among older adults hypertensive patients in the United States was notably greater than that of older adults hypertensive patients in China. Specifically, the highest WWI quartile was 14.79 in the USA compared to 10.91 in China. Our study indicated a high expression of WWI suggesting a decrease in abdominal muscles among the older adults population in the United States (34). Thus, these differences may explain the discrepancy in findings between our study and the Rural Chinese Cohort Study.

### 4.3. Possible mechanism

The mechanisms that underlie the positive correlation between WWI and the prevalence of hypertension in the older adults are of interest. As previously noted, WWI is positively correlated with visceral fat in the body and negatively correlated with abdominal muscle, which suggests that WWI may indicate conditions of excessive fat storage and declining muscle mass (8).

Firstly, muscle mass decreases was closely related to decreased physical activity, increased levels of oxidative stress caused by aging (35). Moreover, the decrease in muscle mass was closely tied to an increase in visceral fat and intramuscular fat, and ultimately results in decreased insulin sensitivity and an increased incidence of cardiovascular events (36). For example, Xia et al.’s (37) research on 2,432 older adults individuals in Shanghai, China, found that sarcopenic overweight/obesity may lead to an increase in the incidence rates and total mortality of cardiovascular diseases in older adults individuals.

Secondly, not only does muscle mass decrease, but the down-regulation of muscle strength and function related to aging-specifically, the reduction in force generated by skeletal muscle per unit area-also increases the risk of hypertension in the older adults (38). These findings may be closely related to the decreased regeneration ability of skeletal muscle cells, decreased mitochondrial function, and an increase in adipocytes in skeletal muscle caused by aging.

Thirdly, the process of aging not only results in changes in the overall amount of body fat, but also causes a redistribution of fat (39). Tankó et al. (40) found that those with high peripheral fat but low abdominal fat had lower blood lipid and blood pressure levels. The storage capacity of fat in peripheral tissues decreases with age, making it difficult for the body to absorb all the free fatty acids. This results in an increase in free fatty acid concentration in the bloodstream, causing excess fat to accumulate in the viscera as visceral fat, leading to inflammation and oxidative stress related to obesity, atherosclerosis, and hypertension (41). Moreover, Guenther et al. (42) found that individuals with more subcutaneous fat had higher concentrations of adiponectin in a study involving 424 Caucasian men and women. These results suggest that there is an inverse correlation between adiponectin levels and visceral fat, with higher concentrations of adiponectin found in individuals with more subcutaneous fat. Adiponectin may help to reduce chronic elevation of blood pressure by reducing inflammation, alleviating oxidative stress damage, and resisting apoptosis. In contrast, the lack of adiponectin can lead to hypertension (43).

Fourthly, as is commonly known, arterial stiffness caused by dysregulation of adipokines can further increase blood pressure with physiological aging (44).

### 4.4. Subgroup analysis

Notably, the positive association in our study appears to be more stable among adults aged between 60 and 69 years. This suggests that WWI may serve as a more reliable indicator of obesity and hypertension risk among younger older adults individuals, but may be less accurate among those of advanced age. Our results are in line with those of Ostchega et al. (30), who reported significantly higher hypertension prevalence rates among individuals with abdominal obesity, except in participants aged 80 years or older. The reasons for this age-dependent difference in the WWI-hypertension association remain unclear and warrant further investigation. The previous study (30) showed that age-adjusted prevalence of abdominal obesity were highest among white individuals (27%), which was relatively consistent with the findings of our present study. Our findings also suggest that low income and education levels may play a significant role in the WWI-hypertension association, as suggested by Kostova et al. (45). Lower socioeconomic status may lead to unhealthy dietary habits, increasing the risk of hypertension. In addition, a lack of education may lead to reduced awareness and screening of hypertension. Sedentary behavior and other unhealthy lifestyle choices also appear to be significant factors in the emergence of hypertension, as documented by Bakker et al. (46). Our study also highlights a possible association between rheumatoid arthritis and hypertension, which is consistent with the findings of Liang et al. (47) Physical inactivity and endothelial dysfunction may be shared pathophysiological factors contributing to these conditions.

### 4.5. Strengths and limitations

This study also has several strengths. Firstly, the previous study (13) only included 650 participants aged over 60 years. In our study, we greatly enlarged the sample size to include 6,674 participants aged over 60 years. This increase in sample size provides sufficient statistical power to detect a positive relationship between the WWI and the prevalence of hypertension. Secondly, the previous study (13) was primarily conducted with East Asian populations, whereas our study has results that can be generalized to the US population as a whole. Thirdly, the previous study (13) primarily focused on a specific population in rural China. In contrast, our study included a diverse sample of 6,674 older adults hypertensive patients across the United States. Fourthly, in Li et al.’s study (13), health data for hypertensive patients aged 75 years and over was not available. Additionally, their age subgroup analysis was limited to hypertensive individuals younger than 60 years and those older than 60 years. In our study, we included hypertensive patients aged 75 years and over. Furthermore, we conducted a thorough analysis of the relationship between WWI and the prevalence of hypertension based on three age subgroups (60–69 years, 70–79 years, and over 80 years). This comprehensive analysis provides additional evidence about the association between the WWI and the prevalence of hypertension. Fifthly, our research results further demonstrate that the positive association between WWI and the prevalence of hypertension is more prominent in patients with a PIR below 2 and sedentary time exceeding 480 min per day. Furthermore, we found a significant association between WWI and hypertension in patients diagnosed with arthritis, which is a novel finding compared to the previous study.

However, this study also has some limitations. Firstly, our cross-sectional design precludes us from establishing causality between WWI and the prevalence of hypertension. Secondly, some of our data are based on self-reported measures, which may be subject to recall bias. Thirdly, although we made adjustments for important covariates in our study, it is important to note that there could be other factors related to hypertension that may affect the connection between WWI and hypertension. Unfortunately, we were unable to adjust for all potential confounders due to the limited information provided by NHANES. This limitation is intrinsic to observational studies based on NHANES data. Therefore, we recommend conducting more extensive longitudinal studies that incorporate detailed data collection to verify our results. It is also recommended that future longitudinal studies investigate a more comprehensive set of variables to solidify these findings. In conclusion, collecting more detailed information in future studies will bolster these findings.

### 4.6. Implication for future researches

According to the research findings of this study, WWI has an advantage in predicting the prevalence of hypertension in younger American older adults individuals. However, the previous Rural Chinese Cohort Study did not explore the positive relationship between WWI and hypertension in Chinese older adults due to the small sample size and the lack of age-stratified analysis for individuals over 60 years old (13). As stated by Li et al. (13), in their research limitations, further large-scale prospective cohort studies are needed to assess the relationship between WWI and incidence of hypertension in older people in China. It is worth noting that, unlike many other parts of the world, China enforces a specific registration system known as the *hukou* system, which restricts internal migration. In China, a person’s *hukou* status, rather than their physical location, determines their access to public services and welfare benefits, including the education system, healthcare, and social security coverage (48). The *hukou* system has created a two-class society with distinct rural–urban divisions (49). Consequently, due to the weak health system, inadequate healthcare human resources, and insufficient government investment in rural areas resulting from the *hukou* system, hypertension awareness, treatment, and control have been consistently found to be lower among rural adults in China (50). This disparity is even worse when it comes to the availability of medical resources for older adults individuals in rural China (51). The findings of this study draw on the conclusions of the NHANES database in the United States, recommending that healthcare providers, public health practitioners, and health policymakers in China should consider the application of non-invasive WWI in older adults Chinese hypertensive patients in rural areas. In the future, Chinese scholars can further explore the relationship between WWI and the prevalence of hypertension in younger older adults individuals through international cooperation.

The current research only focuses on the relationship between hypertension and WWI in older adults individuals in rural China, while ignoring the situation of urban Chinese older adults individuals. A cross-sectional study on global AGEing and adult health (SAGE) conducted by Li et al. (52) across eight provinces in China included a nationally representative subsample of 7,403 respondents aged 60 years and above. The results showed that the prevalence of hypertension and comorbidities was higher among urban participants compared to their rural counterparts. Certain lifestyle behaviors such as sedentary behaviors (53) and high calorie intake (54) contribute to the increasing prevalence of hypertension among urban Chinese older adults individuals. Considering the impact of the *hukou* system in creating a two-class society in China, the relationship between WWI and the prevalence of hypertension among urban Chinese older people still needs further investigation.

Our findings indicate that the association between WWI and the prevalence of hypertension was particularly pronounced in non-Hispanic white individuals, underscoring the significance of considering ethnic differences. These results also highlight the possibility of variations in subgroup findings across different ethnic groups in China. China, as a multi-ethnic country, exhibits variations in geographical distribution, lifestyle habits, cultural heritage, economic status, and genetic background among its diverse ethnic groups. Notably, the prevalence rates of hypertension were significantly higher in the Bouyei ethnic group compared to the Han ethnic group, with these ethnic disparities being more pronounced in the older adults population (55). Additionally, Wang et al. (56) reported a high prevalence of hypertension among rural adults in Xinjiang, China, particularly within the Kazak population. The prevalence rates of hypertension in the Kazak ethnic group were significantly higher than those in the Han ethnic group (52.57% vs. 36.84%). Therefore, further investigations are warranted to analyze the ethnic subgroups of the Chinese population and explore the association between WWI and the prevalence of hypertension in Chinese ethnic minorities.

## 5. Conclusion

Based on our nationwide study, there is a strong correlation between elevated levels of WWI and the risk of developing hypertension among older adults adults. As such, WWI could serve as a unique and valuable biomarker for identifying hypertension risk at an earlier stage in the older adults population.

## Data availability statement

The datasets presented in this study can be found in online repositories. The names of the repository/repositories and accession number(s) can be found in the article/Supplementary material.

## Author contributions

JW and J-HW: conceptualization. JW: methodology, data curation, writing – original draft preparation, and visualization. Q-YY: software and formal analysis. D-jC: validation. YS and Q-ZJ: validation. J-HW: investigation, resources, writing – review and editing, supervision, project administration, and funding acquisition. All authors contributed to the article and approved the submitted version.
