# Supplementary material for: The relationship between obesity associated weight-adjusted waist index and the prevalence of hypertension in US adults aged ≥60 years: a brief report
Source: Front Public Health. 2023 Oct 6;11:1210669. doi: 10.3389/fpubh.2023.1210669 (PMC10587597; doi:10.3389/fpubh.2023.1210669)
Supplement: Supplementary file 1 [file Data_Sheet_1.pdf]

Supplementary Material

The Relationship Between Obesity associated Weight-Adjusted Waist Index and the Prevalence of Hypertension in US Adults Aged⩾60 Years: A Brief Report

Jiao Wang [1], Qing-Ye Yang [1], Dong-jian Chai[1], Yue Su[1], Qi-Zhi Jin[1] and Jin-Hua Wang[1,*]

[1] Department of Cardiology, The Quzhou Affiliated Hospital of Wenzhou Medical University, Quzhou People's Hospital, QuZhou, Zhejiang Province, 324000, P.R. China.

[*] Correspondence: Department of Cardiology, The Quzhou Affiliated Hospital of Wenzhou Medical University, Quzhou People's Hospital, 32400, Min Jiang Road 100, Kecheng District, Quzhou, Zhejiang Province, P.R. China.

E-mail: 15060240206@hnit.edu.cn or wangjinhuazj@126.com

1 Supplementary Figures and Tables

1.1 Supplementary Figures

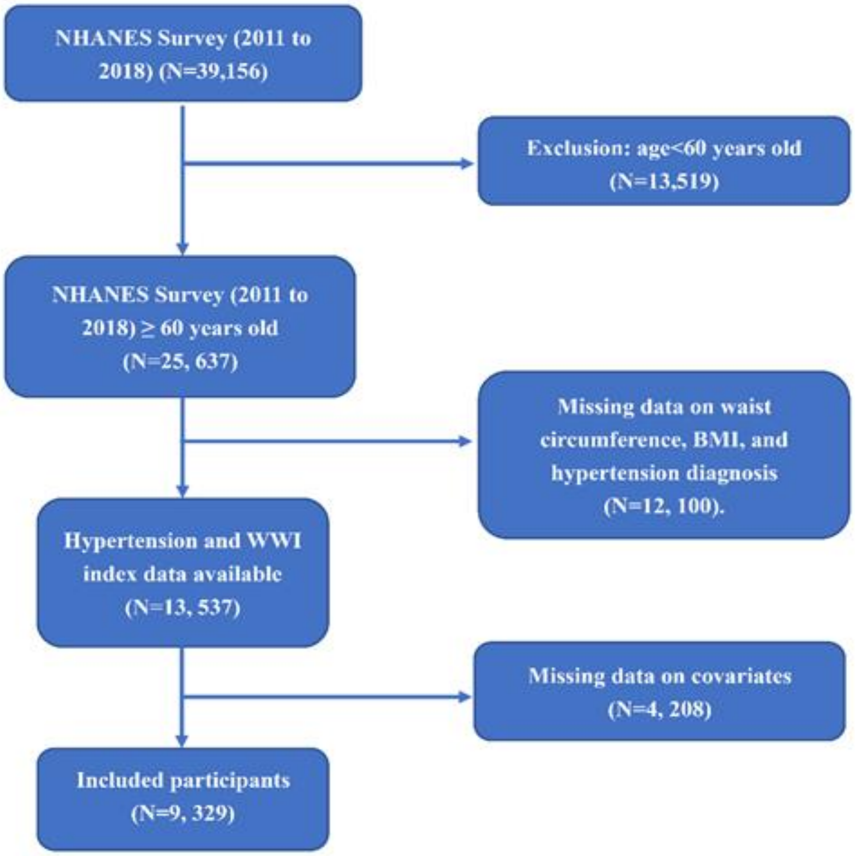

Supplementary Fig.S1. Figure S1 displays a detailed flow chart that highlights the screening process utilized to select eligible participants in NHANES from 2011 to 2018.

1.2 Supplementary Tables

Table S1 Weighted characteristics of elderly participants with or without hypertension

	Overall	Hypertension	Without hypertension	*P* value
N	9329	6674	2655	
**Age (years)**	69.25 ± 6.64	69.97 ± 6.72	67.77 ± 6.24	<0.001
**Gender (%)**				<0.001
Male	45.70	44.09	49.00	
Female	54.30	55.91	51.00	
**Race (%)**				<0.001
Non-Hispanic white	76.74	74.29	81.78	
Non-Hispanic black	8.75	10.58	4.99	
Mexican American	4.10	4.20	3.87	
Other race/ethnicity	10.41	10.92	9.35	
**Level of education (%)**				<0.001
Less than high school	16.23	17.82	12.97	
High school	24.44	26.45	20.29	
More than high school	59.33	55.72	66.75	
**Income to poverty ratio**	3.14 ± 1.58	3.00 ± 1.57	3.41 ± 1.56	<0.001
**Smoking status (%)**				0.003
Never smoker	59.45	59.44	59.46	
Current smoker	9.33	8.43	11.24	
Former smoker	31.22	32.13	29.30	
**Alcohol drinking (%)**				<0.001
Never drinkers	11.97	12.20	11.51	
Former drinkers	25.81	27.09	23.17	
Current drinkers	62.13	60.71	65.32	
**Sedentary behavior (min/d)**	427.48 ± 726.12	445.20 ± 811.90	391.05 ± 504.06	0.023
**Physical activity**				<0.001
Vigorous	13.19	13.52	12.50	
Moderate	33.13	31.62	36.25	
Less than moderate	53.68	54.86	51.25	
**Laboratory examinations**				
Fasting plasma glucose [FPG](mg/dL)	114.71 ± 32.53	117.74 ± 34.37	108.87 ± 27.75	<0.001
	192.67 ± 42.60	129.20 ± 83.01	107.62 ± 64.92	
Triglycerides [TG] (mg/dL)				<0.001
	56.12 ± 17.84	55.02 ± 17.96	58.36 ± 17.38	
High-density lipoprotein cholesterol [HDL-C] (mg/dL)				<0.001
	16.89 ± 6.55	17.46 ± 7.12	15.72 ± 5.00	
Blood urea nitrogen (mg/dL)				<0.001
	0.96 ± 0.44	1.00 ± 0.50	0.90 ± 0.28	
Blood creatinine (mg/dL)				<0.001

**Comorbidities (%)**	20.31	24.75	11.18	
Diabetes diagnosis	9.93	11.92	5.86	<0.001
CVD diagnosis	6.39	7.82	3.44	<0.001
Stroke	50.97	54.31	44.10	<0.001
Arthritis				<0.001
**Medication usage (%)**				
Lipid lowing drugs	84.54	84.98	83.18	**0.141**
Anti diabetes medicine	52.26	53.82	46.34	<0.001
**WWI index**	18.78 ± 1.93	19.31 ± 1.85	18.52 ± 1.92	<0.001
**WWI index categorical**				<0.001
Quartile 1		22.18	37.58	
Quartile 2		24.90	26.73	
Quartile 3		25.65	20.75	
Quartile 4		27.27	14.95	
